# Utilization of xylose by engineered strains of *Ashbya gossypii* for the production of microbial oils

**DOI:** 10.1186/s13068-016-0685-9

**Published:** 2017-01-03

**Authors:** David Díaz-Fernández, Patricia Lozano-Martínez, Rubén M. Buey, José Luis Revuelta, Alberto Jiménez

**Affiliations:** Departamento de Microbiología y Genética, Universidad de Salamanca, Campus Miguel de Unamuno, 37007 Salamanca, Spain

**Keywords:** *Ashbya gossypii*, Xylose, Xylitol, Metabolic engineering, Lipids

## Abstract

**Background:**

*Ashbya gossypii* is a filamentous fungus that is currently exploited for the industrial production of riboflavin. The utilization of *A. gossypii* as a microbial biocatalyst is further supported by its ability to grow in low-cost feedstocks, inexpensive downstream processing and the availability of an ease to use molecular toolbox for genetic and genomic modifications. Consequently, *A. gossypii* has been also introduced as an ideal biotechnological chassis for the production of inosine, folic acid, and microbial oils. However, *A. gossypii* cannot use xylose, the most common pentose in hydrolysates of plant biomass.

**Results:**

In this work, we aimed at designing *A. gossypii* strains able to utilize xylose as the carbon source for the production of biolipids. An endogenous xylose utilization pathway was identified and overexpressed, resulting in an *A. gossypii* xylose-metabolizing strain showing prominent conversion rates of xylose to xylitol (up to 97% after 48 h). In addition, metabolic flux channeling from xylulose-5-phosphate to acetyl-CoA, using aheterologous phosphoketolase pathway, increased the lipid content in the xylose-metabolizing strain a 54% over the parental strain growing in glucose-based media. This increase raised to 69% when lipid accumulation was further boosted by blocking the beta-oxidation pathway.

**Conclusions:**

*Ashbya gossypii* has been engineered for the utilization of xylose. We present here a proof-of-concept study for the production of microbial oils from xylose in *A. gossypii*, thus introducing a novel biocatalyst with very promising properties in developing consolidated bioprocessing to produce fine chemicals and biofuels from xylose-rich hydrolysates of plant biomass.

**Electronic supplementary material:**

The online version of this article (doi:10.1186/s13068-016-0685-9) contains supplementary material, which is available to authorized users.

## Background

The implementation of novel technologies to produce more sustainable and clean oil-based fuels and chemicals is an important challenge for the industrial biotechnology field. In this context, the use of non-edible oils such as microbial oils represents a sustainable alternative for the production of functional oils and hydrocarbon-based compounds [1].

Microbial oils have several advantages over other oil resources: the fermentative processes are independent of climate and, more importantly, the use of either waste industrial by-products or plant biomass as substrates for microbial fermentation avoids competition with edible resources and makes the process environmentally friendly [2]. In this regard, the development of novel microbial biocatalysts with different properties in terms of substrate utilization, fermentation conditions, and broad-range compound production is required. Therefore, it is necessary to engineer widely used industrial microorganisms such as bacteria, yeast, and fungi for the efficient utilization of low-cost substrates that can be used for the production of biofuels and other oleochemicals [3–5].

*Ashbya gossypii* is a filamentous hemiascomycete that is extensively used for the industrial production of riboflavin [6–9]. The use of *A. gossypii* in industry is considered a paradigm of sustainable white biotechnology for the microbial production of riboflavin and other vitamins. Importantly, a large number of genomic, bioinformatic, and biotechnological tools are available for *A. gossypii* [10–12], thus allowing the development of systems metabolic engineering approaches to industrial applications of the fungus. The use of *A. gossypii* for microbial fermentation presents other biotechnological advantages, such as the ability to grow using industrial by-products and low-cost oils, the partial autolysis of its hyphae at late growth phases, and the harvesting of the mycelia by simple filtration [3].

Recently, we have reported engineered strains of *A. gossypii,* which are able to accumulate up to 70% of their cell dry weight (CDW) as lipid content. This was achieved using a multigene approach consisting of both the heterologous overexpression of the ATP-citrate lyase (ACL) activity from *Yarrowia lipolytica* and the inactivation of the endogenous lipid beta-oxidation pathway by *POX1* gene deletion [13]. Metabolic engineering has also been applied to both the fatty acid elongase and desaturase systems with a view to generating novel *A. gossypii* strains that are able to accumulate high-value oil-related compounds. For example, engineered *A. gossypii* strains lacking both very long chain fatty acids and polyunsaturated fatty acids, which are undesired features in biodiesel blends, have been described [14].

**Fig. 1:**
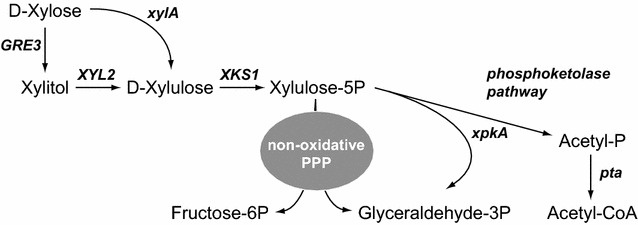
Schematic pathway for the utilization of xylose. *GRE3*, xylose reductase; *xylA*, xylose isomerase; *XYL2*, xylitol dehydrogenase; *XKS1*, xylulose kinase; *xpkA*, X5P phosphoketolase; *pta*, phosphotransacetylase

The *A. gossypii* industrial fermentations are currently achieved using low-cost plant oils as carbon source. However, as mentioned above, the use of non-edible substrates, such as plant biomass, for microbial bioconversion is gaining much attention during recent years [15]. Therefore, it is required to explore the ability of *A. gossypii* to metabolize C5 sugars, such as d-xylose, present in hydrolysates of plant biomass. So far, it is described that xylose as the only carbon source cannot support the growth of *A. gossypii*, though it can be converted to xylitol, thus suggesting that *A. gossypii* is equipped both with transporters for pentose sugars and the catalytic machinery for the conversion of d-xylose into xylitol [16]. In this regard, two pathways for the utilization of xylose have been described (Fig. 1): in bacteria and some anaerobic fungi, the conversion of d-xylose to d-xylulose is catalyzed by a xylose isomerase (XI, *xylA*); in yeast, fungi and other eukaryotes the transformation of d-xylose into d-xylulose is accomplished by a two-step pathway which involves both a xylose reductase (XR, *XYL1*, *GRE3*) and a xylitol dehydrogenase (XDH, *XYL2*) [15, 17]. d-xylulose can subsequently enter the non-oxidative pentose phosphate pathway (PPP) after being phosphorylated by xylulose kinase (XK, *XKS1*) into xylulose-5-phosphate (X5P).

Two general strategies have been employed for the construction of xylose-utilizing yeast strains: the overexpression of a bacterial XI along with XK, and the overexpression of a complete XR-XDH-XK pathway [18, 19]. Strains with engineered xylose metabolism combined with additional manipulations have also been described for the production of different high-value chemicals [19]. For example, a recombinant phosphoketolase pathway, which directly channels the X5P carbon flux toward acetate/acetyl-CoA synthesis, has been used for the production of fatty acids and ethanol [20, 21].

Here we describe the development of novel *A. gossypii* strains that have been engineered for the utilization of xylose as carbon source. The overexpression of the native XR-XDH-XK pathway permits *A. gossypii* to grow using xylose as the only carbon source. In addition, further strain engineering using a heterologous phosphoketolase pathway along with the abolition of the beta-oxidation pathway resulted in the isolation of *A. gossypii* strains which are able to produce a high yield of biolipids from xylose as the only carbon source. In sum, we describe a novel microbial biocatalyst, which can be useful for the production of higher added-value lipids, fine chemicals, and biofuels from xylose-rich biomass. The biotechnological significance and the future applications of these strains are further discussed.

## Results

### Identification of the xylose utilization pathway in *A. gossypii*

*Ashbya gossypii* is able to accumulate xylitol when xylose is used as the carbon source [16], suggesting that a metabolic pathway for xylose utilization must exist in this fungus. Indeed, a putative XR-XDH-XK pathway for xylose utilization in *A. gossypii* was found at the KEGG Pathway database (http://www.genome.jp/kegg/pathway.html). The sequences of the predicted XR (ACL107Cp), XDH (ABR229Cp), and XK (AGR324Cp) enzymes were obtained and a BLASTP analysis was carried out. The *ACL107C* gene resulted in a syntenic ortholog of the *S. cerevisiae GRE3* gene that codes for an aldose reductase. The predicted sequence of the ACL107Cp showed high identity (60–65%) with XR enzymes from Saccharomycetaceae yeast species such as *Zygosaccharomyces rouxii*, *Candida tropicalis*, or *C. dubliniensis*. The *ABR229C* gene showed homology with the *S. cerevisiae XYL2* and other XDH-coding orthologs such as *XYL2* from *Scheffersomyces (Pichia) stipitis*. Likewise, the AGR324C protein showed high similarity (55–65%) with both yeast and fungi XK enzymes from species such as *Saccharomyces cerevisiae*, *S. stipitis*, and *Neurospora crassa*. Consequently, the *A. gossypii ACL107C*, *ABR229C,* and *AGR324C* genes were termed as *GRE3*, *XYL2*, and *XKS1*, respectively, due to their homology with the *S. cerevisiae* orthologs.

### Overexpression of the xylose utilization pathway in *A. gossypii*

*Ashbya gossypii* cannot grow on xylose as the only carbon source, even though a putative XR-XDH-XK pathway was identified to be encoded in its genome. Gene overexpression of either native or heterologous xylose assimilation pathways (XI-XK or XR-XDH-XK) has been successfully used for the generation of different bacterial or yeast strains able to metabolize xylose [15]. Therefore, we wished to analyze whether boosting the native pathway for xylose utilization (XR-XDH-XK) can improve the ability of *A. gossypii* to grow on xylose as the only carbon source.

**Fig. 2:**
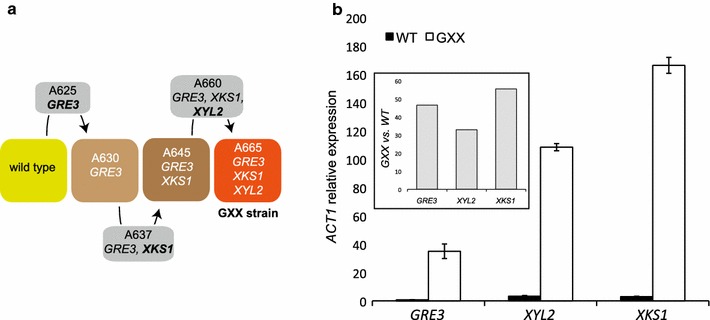
Overexpression of the endogenous xylose utilization pathway in *A. gossypii*. **a** Pipeline of the construction of the GXX strain. The expression of the Cre recombinase enabled the *loxP*-*KanMX*-*loxP* marker to be eliminated (from the* gray*-colored strains) and subsequently reused. **b** Quantitative real-time PCR of the *GRE3*, *XYL2*, and *XKS1* genes in the WT and GXX strains of *A. gossypii*. Relative transcription levels of the genes after 48 h of culture in MA2-rich medium. Transcription levels were normalized using the *A. gossypii ACT1* gene as a reference. The results are the means of two independent experiments performed in duplicate and are expressed as a ratio of the cDNA abundances of the target genes with respect to the *ACT1* mRNA levels. The *inset* represents the overexpression fold of the genes in the GXX strain with respect to the WT strain

The generation of a triple (*GRE3*, *XYL2*, and *XKS1*) overexpressing strain was achieved after three rounds of transformations, as depicted in Fig. 2a. The overexpression of the genes *GRE3*, *XYL2*, and *XKS1* in the triple mutant strain (A665-GXX strain) was confirmed by qRT-PCR (Fig. 2b). All three genes showed mRNA levels in the GXX strain 30- to 50-fold higher than in the wild-type strain.

**Fig. 3:**
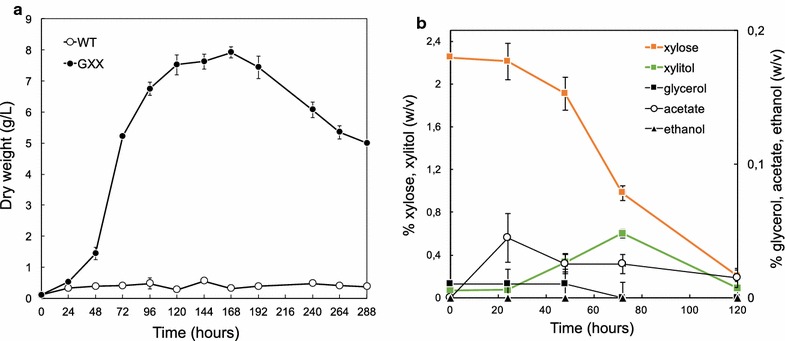
Utilization of xylose by the GXX strain of *A. gossypii*. **a** Biomass production of the WT and GXX strains grown in MA2 medium with 2% xylose as the only carbon source. **b** Xylose consumption and metabolite production by the GXX strain grown in MA2 medium with 2% xylose as the only carbon source

The ability of the GXX strain in utilizing xylose was evaluated in flask cultures during 12 days using MA2-rich media with 2% xylose as the only carbon source. The GXX strain showed exponential growth (specific growth rate µ_xyl_ = 0.0537 h^−1^) during the first 72 h of culture in xylose-based media (Fig. 3a). Unexpectedly, the GXX strain was able to produce more biomass in xylose-based media (7–8 g/L of biomass) than in glucose-based media (6–7 g/L of biomass) (Fig. 3a; Additional file 1). In addition, we found that the germination of the spores from the GXX strain was significantly delayed in xylose-based media compared to glucose-based media (Fig. 3a; Additional file 1). Accordingly, the consumption of xylose was very low until sufficient biomass was reached in the culture (Fig. 3a, b). The specific consumption rate of xylose during the exponential growth of the GXX strain in MA2-rich media with 2% xylose was 0.226 mmol/g/h. Excretion of xylitol to the culture media occurred soon after the xylose started to be consumed. The concentration of xylitol in the supernatant reached 6 g/L after 72 h of culture, but it was subsequently consumed (Fig. 3b). Excretion of other metabolites such as glycerol, acetate, and ethanol was very low (Fig. 3b).

**Fig. 4:**
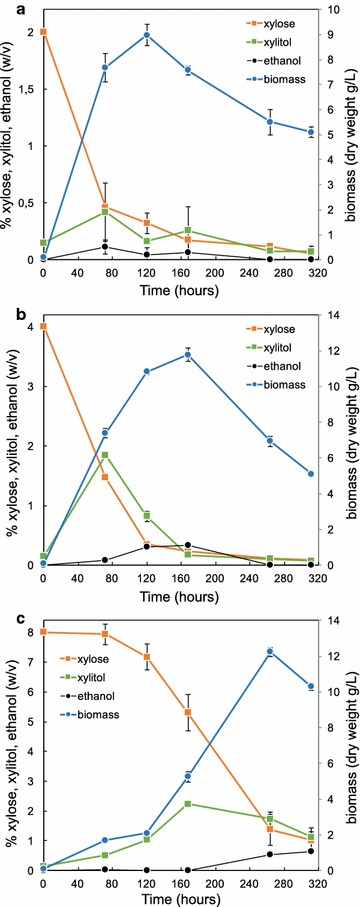
Growth kinetics of the GXX strain of *A. gossypii* with different concentrations of xylose. Biomass production (*orange line*), xylose consumption, xylitol and ethanol production of the GXX strain grown in MA2 with 2% xylose (**a**), 4% xylose (**b**), and 8% xylose (**c**) as the only carbon source

Increasing concentrations of xylose (2, 4, and 8%) were used to further analyze the GXX growth abilities. MA2-rich media with 4% xylose increased significantly the production of biomass without changing the growth kinetics of the GXX strain (Fig. 4a, b). In contrast, the use of MA2-rich media with 8% xylose resulted in a strong delay of the culture for biomass production and an extended trophic phase, which agrees with a lack of xylose consumption during the first 72 h of culture (Fig. 4c). Nevertheless, the production of biomass was maximal with 8% xylose after 264 h of culture, reaching more than 12 g/L of CDW (Fig. 4c).

Interestingly, the excretion on xylitol was highest when 1.5–2.5% of xylose was consumed in all conditions that were analyzed, reaching very high concentrations in the culture media (up to 22.6 g/L from 8% xylose media); yet again the excreted xylitol was consumed afterwards (Fig. 4). Ethanol excretion (3–6 g/L) was also detected with 4 and 8% xylose, and the levels of ethanol in the culture media were highest when most of the xylose was consumed (Fig. 4b, c).

**Fig. 5:**
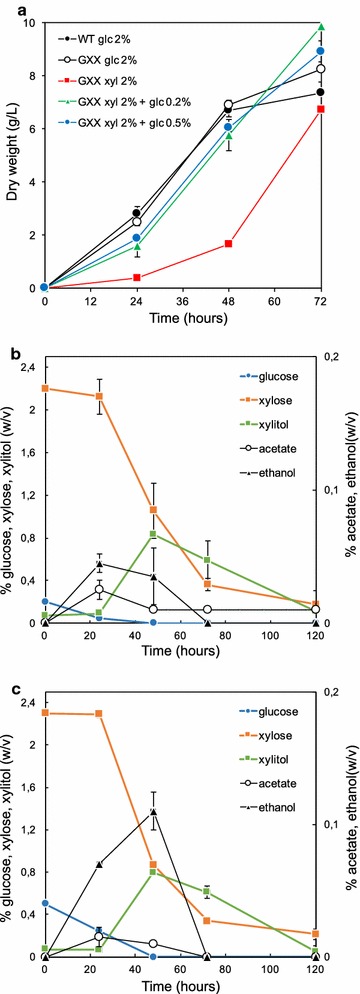
Growth kinetics of the GXX strain of *A. gossypii* using mixed sugars. **a** Biomass production of the WT and GXX strains at early stages of cultures using different sugar formulations. **b**, **c** Sugar consumption and metabolite production of the GXX strain grown in MA2 with 2% xylose plus 0.2% glucose (**b**) and 2% xylose plus 0.5% glucose (**c**) as the carbon sources

As mentioned above, the germination of the spores and, hence, the initiation of the exponential growth was significantly delayed in xylose-based media in comparison with the glucose-based cultures (Fig. 3a; Additional file 1). While MA2 glucose-based media produced high levels of biomass soon after the first 6–8 h, the cultures in MA2 xylose-based media were delayed in the production of biomass. This phenotype was almost completely restored by the addition of 0.2–0.5% glucose to the xylose-based culture media (Fig. 5a). Accordingly, the xylose began to be consumed earlier during the culture when most of the glucose was exhausted (Fig. 5b, c). Again, a peak of xylitol excretion to the culture media (8–8.3 g/L) occurred when about 10 g/L of xylose were consumed (48 h of culture). Certain levels of ethanol (up to 1 g/L), produced by fermentation of glucose, were detected in the culture media during the early time-points of the culture, but this ethanol was latter consumed (Fig. 5b, c).

Overall, our results demonstrate that the overexpression of the XR-XDH-XK pathway in *A. gossypii* allows the utilization of xylose and supports growth in xylose-based culture media.

### Heterologous overexpression of a phosphoketolase pathway in the GXX strain

The overexpression of the XR-XDH-XK pathway in *A. gossypii* allows channeling the carbon flux from xylose, through X5P, toward the pentose–phosphate pathway. However, our aim in this work was to redirect carbon flux to the production of lipids. Enzymatic activities of the “so called” phosphoketolase pathway are able to catalyze the transformation of X5P into acetyl-CoA (Fig. 1), which is the essential donor molecule for fatty acid (FA) biosynthesis. In this regard, the overexpression of a recombinant phosphoketolase pathway, including the phosphotransacetylase (*pta*) and X5P phosphoketolase (*xpkA*) from *Bacillus subtilis* and *Aspergillus nidulans*, respectively, has been used for the production of fatty acid ethyl esters from glucose [20]. Therefore, we next decided to overexpress both a X5P phosphoketolase and a phosphotransacetylase in the GXX strain in order to redirect carbon flux to the biosynthesis of acetyl-CoA.

The ORFs of the *B. subtilis pta* and *A. nidulans xpkA* genes were used for the construction of two overexpression cassettes using the strong promoter P_*AgGPD*_. All fragments for each overexpression module were assembled following a one-pot DNA-shuffling method (see Additional file 2 and “Methods” section for details). The overexpression cassette for the *pta* gene was targeted to the *ADR304W* locus, while the overexpression cassette for the *xpkA* gene was inserted into the *AGL034C* locus (Additional file 2). The disruption of either *ADR304W* or *AGL034C* does not affect growth in *A. gossypii*, as previously described [13, 14]. The integration of the overexpression cassettes in the target genomic loci of the GXX strain was achieved after two rounds of transformations and it was confirmed by analytical PCR and DNA sequencing.

To confirm the transcription of both *pta* and *xpkA*, total mRNA of the new strain (A729, GXX-PX strain), growth in MA2 rich media with 2% xylose, was analyzed by qRT-PCR. The levels of the *pta* and *xpkA* mRNAs were 50- and 10-fold higher than those of the *UBC6* housekeeping gene taken as a reference, respectively. With regard to the xylose utilization capacities, we did not find differences in growth kinetics between the GXX-PX strain and its parental strain GXX in MA2-rich media with either glucose or xylose as the carbon sources.

### Lipid production from xylose in engineered strains of *A. gossypii*

**Fig. 6:**
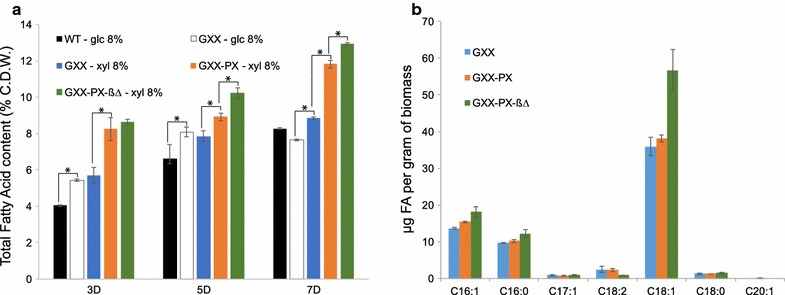
Lipid production of the *A. gossypii* engineered strains. **a** Lipid quantification of the WT and the engineered strains grown during 3, 5 and 7 days in media containing the indicated carbon sources. The results are the means of two independent experiments performed in duplicate. The *error bars* represent the standard deviations. The Student’s *t* test was performed to determine significant differences (*P* < 0.05). **b** Lipid profiles of the engineered strains grown during 5 days in media containing 8% xylose

We wished to further investigate the lipidogenic capacities of the modified strains GXX and GXX-PX. According to the results shown above, MA2 media with 0.2% glucose plus 8% xylose were used to analyze the total lipid production of the strains GXX and GXX-PX. Lipid content was measured at 3, 5, and 7 days of culture in order to analyze the accumulation of lipids both during the exponential, early productive, and late productive phases of *A. gossypii* culture. MA2 media with 8% glucose were also used as a reference of lipid production of both the wild-type strain and the GXX strain. Surprisingly, the GXX strain showed a 20–40% higher lipid production than the wild-type during the 3–5 days of culture when grown either in glucose or xylose media (Fig. 6a). In addition, the GXX-PX strain showed significantly higher levels of lipid production from xylose, reaching a 12% of CDW after 7 days of culture, thus demonstrating that carbon flux was efficiently channeled toward lipid synthesis in this strain (Fig. 6a).

**Fig. 7:**
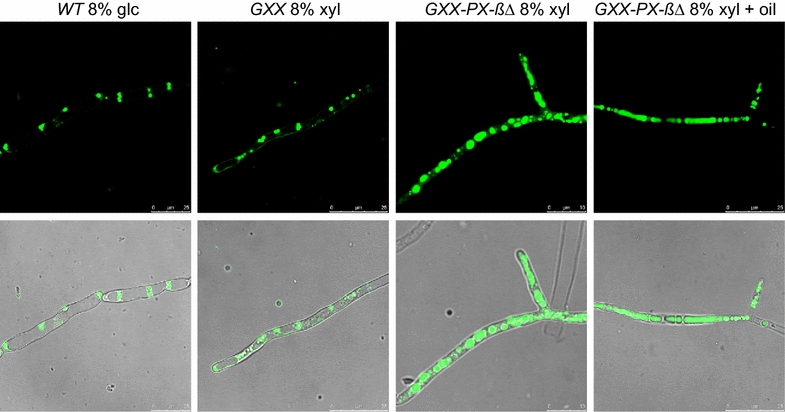
Lipid bodies of the *A. gossypii* engineered strains. Micrographs of the WT and the engineered strains grown during 5 days in media containing different carbon sources. 2% oleic acid was added when culture media was supplemented with oil. Lipid bodies were stained with Bodipy and visualized under fluorescence microscopy

Lipid accumulation in *A. gossypii* can be further increased through the blocking of the beta-oxidation pathway [13]. Accordingly, the *POX1* gene was deleted in the GXX-PX strain, and the lipid production was measured. The new engineered *GXX*-*PX*-*pox1∆* (*GXX*-*PX*-*ß∆*) strain showed the highest levels of lipid production all along the culture time-points, reaching a 13% of total fatty acids in the CDW after 7 days of culture in 8% xylose-based media, which represents an increase of 69% with respect to GXX strain grown in 8% glucose-based media (Fig. 6a). The lipid profiles of the three xylose-metabolizing strains revealed that the *GXX*-*PX*-*ß∆* strain accumulates significantly higher concentrations of oleic acid (C18:1) than the *GXX* and *GXX*-*PX* strains, which showed no differences among them. The production of lipids in the engineered strains could be visualized directly by bodipy staining of the intracellular lipid bodies. As shown in the Fig. 7, both the number and the size of lipid bodies in the *GXX*-*PX*-*ß∆* strain were significantly larger than those in the GXX and wild-type strains grown in xylose and glucose, respectively. In addition, the supplementation of the xylose-based media with 2% oleic acid resulted in a huge accumulation of intracellular lipids (Fig. 7; Additional file 3). Hence, our results demonstrate that *A. gossypii* can be engineered for the utilization of xylose and lipid production.

## Discussion

Xylose-rich feedstocks and by-products are between the most abundant and low-priced substrates with biotechnological application. Therefore, the assimilation of xylose by industrial biocatalysts is an important issue that can help microbial fermentations to be more economically feasible and, consequently, the optimization of pentose utilization along with the design of novel microbial tools is required [19]. In this work, we have developed novel strains of *A. gossypii* that can utilize xylose as the only carbon source. Furthermore, metabolic flux from xylose has been channeled for the production of biolipids by the heterologous expression of a phosphoketolase pathway in *A. gossypii*.

Although the GXX strain and derivatives can reach equal biomass titers using either glucose or xylose, the use of glucose is preferred when both sugars are present in the culture media. Indeed, glucose (0.2%) is essential to avoid a delay in the germination of spores of *A. gossypii*. This may reflect a general regulatory mechanism of the C5/C6 sugar uptake by carbon catabolite repression, which eventually would interfere with co-fermentation of mixed sugars, as described for most bacteria and eukaryotes [15]. Therefore, the optimization of xylose uptake using engineered xylose-specific transporters can improve the simultaneous consumption of mixed sugars [15, 22–25].

In spite of the sequential utilization of xylose by the *A. gossypii* engineered strains, we could detect high concentrations of xylitol in the culture media that was eventually consumed, which indicate both a high conversion rate of xylose to xylitol and an active mechanism of xylitol excretion/uptake in *A. gossypii*. The transient accumulation of xylitol indicates that the XDH activity is a rate-limiting step during xylose assimilation. This might be explained by the possible effect of a decreased oxygenation of the culture when biomass becomes high. Indeed, the oxygen availability and aeration conditions were shown to affect the enzyme activities for xylitol production in different xylose-utilizing microorganisms [26–28]. Alternatively, the XDH enzyme might become saturated by an excess of the substrate, thus triggering an increase of the excretion of xylitol. The high concentration of xylitol in the culture media (22.9 g/L in 8% xylose media, Fig. 4c) has a prominent biotechnological interest, since *A. gossypii* can be also considered a potential microbial factory for the industrial production of xylitol. Thus, depending of the composition of the culture media, yields between 0.5 and 0.97 g of xylitol per gram of xylose were measured.

We have previously reported engineered *A. gossypii* strains that are able to accumulate high levels of lipids in oil-based culture media [13]; however, when sugar-based substrates are used, the lipid biosynthesis capacity of microbial catalysts is often hindered by regulatory mechanisms controlling the de novo lipid synthesis [29, 30]. The overexpression of a phosphoketolase pathway in our *GXX*-*PX* strain induced an increase of lipid synthesis, thus demonstrating that metabolic flux is channeled from X5P toward the synthesis of acetyl-CoA. This strategy was previously used for the production of fatty acid ethyl esters in *S. cerevisiae* [20]. Also, although an additional increase in lipid synthesis is obtained by blocking the beta-oxidation oxidation pathway in the *GXX*-*PX*-*ß∆* strain, our results suggest that the lipid degrading activity of this pathway may not be very high in the *GXX*-*PX* strain under the conditions assayed (8% xylose).

Strikingly, the GXX strain produced more lipids than the wild-type in glucose-based media during the 3–5 days of culture. The presence of a constitutively active XR-XDH-XK pathway in the GXX strain might affect the activity of the PPP, which has been shown to metabolize a 30% of the glucose-6-P pool in *A. gossypii* [31]. In turn, an increase of the PPP in the GXX strain might contribute to increase the generation of reducing power in the form of NADPH, which eventually triggers a higher lipid production. Indeed, the reducing power of NADPH/NADH has been shown to play a determinant role in the fatty acid synthesis in oleaginous fungi [32].

Other reports have described genetic customization of microorganisms for the production of lipids from xylose. In the oleaginous yeast *Y. lipolytica*, the heterologous expression of the oxidoreductase pathway (XR-XDH) from *S. stipitis* in combination with other manipulations have been described in two recent works: Ledesma-Amaro et al. [33] have shown the combination of XR-XDH from *S. stipitis* with the overexpression of the endogenous XK in a lipid overproducer genetic background, thus obtaining a 35% of CDW in lipid accumulation under controlled bioreactor conditions; besides, Li and Alper [34] also described the use of XR and XDH from *S. stipitis* along with adaptive-evolutionary engineering in a strain which is able to accumulate 15 g/L of lipids in bioreactor fermentations. In addition, lipid production from xylose has also been described in oleaginous yeast that are able to naturally utilize xylose such as *Rhodosporidium toruloides* and *Mortierella isabellina* with lipid titers of 9.5 and 18.5 g/L, respectively [35, 36]. While *R. toruloides* was engineered to overexpress the lipogenic genes *ACC1* and *DGA1* [36], culture conditions were optimized for lipid accumulation from xylose in *M. isabellina* [35]. In comparison, our engineered strains showed less capacities for lipid accumulation (9–13% of CDW) in the conditions assayed; however, it is worthy to mention that the use of *A. gossypii* presents biotechnological advantages regarding downstream processing and genome engineering, which allow a significant room for improvement of the xylose-utilizing strains.

Lipid titers in our engineered strains may also be constrained by mechanisms affecting the biosynthesis of fatty acids such as feedback inhibition and cofactor imbalance. In this regard, it has been described that acyl-CoA esters regulate the activity of the fatty acid synthase and the acetyl-CoA carboxylase in *S. cerevisiae* [29, 37]. Hence, customized microorganisms lacking metabolic bottlenecks for lipid production have been described, providing evidence that rewiring the de novo lipid regulation can increase the conversion yields of carbohydrates to lipids [30, 37–39]. Further work is foreseen in *A. gossypii* to improve the conversion of carbohydrates to lipids either by engineering the regulators of lipid synthesis or introducing additional lipidogenic manipulations. Indeed, our recent results anticipate that feedback regulation of lipogenic genes exerted by the acyl-CoA pool in *A. gossypii* can be abrogated using engineered alleles of the *MGA2* gene (unpublished results).

In this work, flask cultures with controlled concentrations of xylose in rich media have been performed. However, the exploitation of xylose-rich resources also requires the identification of the critical parameters affecting fermentation productivity. In this regard, the optimization of the culture conditions and downstream processing in a controlled bioreactor most likely would increase titer, yield, and productivity of the xylose-utilizing engineered strains of *A. gossypii*.

## Conclusions

We present here a proof-of-concept study demonstrating the undertaking potential of *A. gossypii* as a competitive biocatalyst for the industrial production of biolipids from xylose. The importance of the present work relies on the feasibility of *A. gossypii* as a cell factory, which enables the application of systems metabolic engineering, fluxomics, and model-based approaches for the generation of improved strains with broad-range abilities for microbial fermentations. A large number of applications for the use of xylose-rich lignocellulosic feedstocks are being recently reported such as the production of ethanol, butanol, butanediol, hexadecanol, and organic acids [40–43]. Hence, it is worthy to mention that enabling *A. gossypii* to use xylose as the only carbon source opens new opportunities for the harnessing of xylose-rich substrates not only for the production of microbial oils, but also a wide range of high-value industrial products such as fine chemicals, riboflavin and other vitamins, purines, and xylitol.

## Methods

### *Ashbya gossypii strains and growth conditions*

The *A. gossypii* ATCC 10895 strain was used and considered a wild-type strain. Other *A. gossypii* strains used in the study are listed in Additional file 4. *Ashbya gossypii* cultures were initiated with spores (10^6^ spores per liter) and carried out at 28 °C in MA2-rich medium using either glucose and/or xylose as carbon sources at the indicated concentrations [6]. *Ashbya gossypii* transformation, its sporulation conditions, and spore isolation were as described previously [6, 44]. Concentrations of 250 mg/L for geneticin (G418) (Gibco-BRL) were used where indicated.

### Gene overexpression and gene deletion

Different transformation cassettes were used either for the overexpression of endogenous genes (i.e., *GRE3*, *XYL2*, and *XKS1*), the overexpression of heterologous genes (i.e., *pta* and *xpkA*) or gene deletion.

For the overexpression of endogenous genes, the promoter sequence of the *AgGPD* gene was integrated upstream of the ATG initiator codon of each gene. Overexpression cassettes comprising the *AgGPD* promoter (*P*_*AgGPD*_) and the *loxP*-*KanMX*-*loxP* selectable marker, conferring resistance to G418, were PCR-amplified using specific primers for each gene (Additional file 5).

For the overexpression of heterologous *pta* and *xpkA* genes, each open reading frame was PCR-amplified using specific primers for each gene (Additional file 5). The *pta* ORF was amplified from *Bacillus subtilis* genomic DNA and the *xpkA* ORF was amplified from the plasmid pMPa (Dr. Jens Nielsen), which has been described elsewhere [20]. The overexpression modules comprised two recombinogenic flanks, a selection marker *loxP*-*KanMX*-*loxP*, and the corresponding ORF with both promoter and terminator sequences (Additional file 2). For the overexpression of the *pta* gene, recombinogenic flanks targeting the *ADR304W* locus were used, and the regulatory sequences were the promoter of *AgGPD* and the terminator of *AgPGK1*. For the overexpression of the *xpkA* gene, recombinogenic flanks targeting the *AGR034C* locus were used, and the regulatory sequences were the promoter of *AgGPD* and the terminator of *AgENO2.* All fragments for each overexpression module were PCR-amplified (see Additional file 5 for primer sequences), verified by DNA sequencing and assembled following a one-pot DNA-shuffling method using the sequence of the *Bsa*I restriction enzyme in the acceptor vector, as previously described [45]. The overexpression modules were finally isolated by enzymatic restriction with *Sap*I.

For the deletion of *AgPOX1*, a gene replacement cassette was constructed for the *POX1* gene by PCR amplification of the *loxP*-*KanMX*-*loxP* marker (see primer sequences in Additional file 5).

Spores of *A. gossypii* were transformed with the corresponding overexpression/deletion cassettes, and positive clones were selected in G418-containing medium. Homokaryon clones were obtained by sporulation of the primary transformants. The correct genomic integration of each overexpression/deletion cassette was confirmed by analytical PCR followed by DNA sequencing. Gene overexpression was further analyzed by qRT-PCR. The *loxP* repeated inverted sequences present in the *loxP*-*KanMX*-*loxP* marker enabled the selection marker to be eliminated and subsequently reused by expressing a Cre recombinase, as described elsewhere [13].

### Quantitative real-time PCR

Quantitative real-time PCR (qRT-PCR) was performed with a LightCycler 480 real-time PCR instrument (Roche), using SYBR Green I master mix (Roche) and following the manufacturer’s instructions. Total RNA samples were obtained as described previously [9], and cDNA samples were prepared using the Transcriptor First Strand cDNA Synthesis Kit (Roche). Primer sequences are indicated in Additional file 5. All real-time PCR reactions were performed in duplicate and in at least two independent experiments. Quantitative analyses were carried out using the LightCycler 480 software. The mRNA level of the target genes was normalized to that of *AgUBC6* and was calculated using the 2^−∆∆Ct^ method [46].

### HPLC analysis of metabolites

Glucose, xylose, xylitol, ethanol, glycerol, and acetate from culture supernatants were analyzed with a Waters Alliance 2795 High-performance liquid chromatography system equipped with a REZEX ROA Organic Acid H+ (8%) column coupled to RI detector (Waters 410). The mobile phase was 0.005 N H_2_SO4 and the flow rate was 0.6 mL/min. All samples were filtered through 0.45 µm filters and 25 μL of each sample were injected.

### Lipid extraction for gravimetric quantitative analysis

Lipids were extracted from 50 mL flask cultures grown in MA2-rich media with carbon sources at indicated concentrations. The cultures were initiated from an overnight pre-inoculum and were incubated for 3–7 days at 28 °C and 200 r.p.m. The mycelium biomass was collected by filtration, lyophilized and the dry cell weight of each sample was determined. Extraction with chloroform/methanol was performed by applying a modification of Folch’s method [47, 48]; equal volumes of methanol and chloroform were added to the mycelium powder and mixed vigorously by vortex. Then ½ volume of H_2_O was added and mixed again. After centrifuging for 5 min at 2000 r.p.m., the lower organic phase was collected and the total fatty acids content was determined gravimetrically after evaporation of organic solvents.

### Lipid extraction for gas chromatography and mass spectrometry analyses

Lyophilized biomass was resuspended in 1 mL of methanol and sulfuric acid (97.5% methanol and 2.5% sulfuric acid) with an internal standard. The samples were incubated at 80 °C for 1.5 h. The reaction that takes place is a transesterification of triglycerides with methanol in presence of an acid catalyst. This reaction is necessary for the subsequent detection of methyl esters of fatty acids in the gas chromatograph. The reaction was stopped by the addition of 1.5 mL H_2_O. Then 0.45 mL of hexane was added and the mixture was vigorously stirred. The upper phase was recovered after centrifugation for 5 min to 2500 r.p.m. 100 μL was collected and placed in glass vials for subsequent analysis. Methyl esters of fatty acids dissolved in hexane were analyzed on a gas chromatograph coupled to a mass spectrometer (GC–MS). GC–MS was carried out using the GC17 Shimazdy gas chromatograph and Shimazdy QP5000 mass spectrometer. A column DB-5 (30 m long, 0.25 mm internal diameter and 25 μm of film) was used. The conditions for the analysis were as follows: it was used helium with a flow of a 1.3 mL/min as a carrier gas, with a Split-ratio 60:1. The injector temperature was 270 °C and the interface temperature was 290 °C. The oven followed this program: initial temperature of 90 °C for 5 min, a ramp of 12 °C/min to 190 °C, and a ramp of 4 °C/min to 290 °C. The fatty acids were identified by comparison with the methyl esters of fatty acids of standard commercial sample (FAME32; Supelco). The total quantification of fatty acids was carried out following the method of standard internal pattern using 50 μg of heptadecanoic acid C17:0 (Sigma).

## Additional files

**Additional file 1.** Utilization of glucose by the GXX strain of *A. gossypii.* Biomass production of the WT and GXX strains grown in MA2 medium with 2% xylose as the only carbon source (upper panel). Glucose consumption and metabolite production by the WT (middle panel) and GXX strains (lower panel) grown in MA2 medium with 2% glucose as the only carbon source.

**Additional file 2.** Cloning strategy for the overexpression of *pta* and *xpkA* genes. (A) Six modules were assembled for the construction of the overexpression cassettes: integration modules 5′ and 3′, selection module, promoter, terminator and the corresponding ORF (*pta* or *xpkA*). (B) The six modules were assembled following a one-pot DNA-shuffling (see “Methods” section) and the overexpression cassettes were integrated in the corresponding genomic loci (*ADR304W* for the *pta* cassette and *AGR034C* for the *xpkA* cassette).

**Additional file 3.** Lipid bodies of the *A. gossypii GXX*-*PX*-*ß∆* strain. Micrograph of the *GXX*-*PX*-*ß∆ strain* grown during 5 days in media containing 8% xylose plus 2% oleic acid. Lipid bodies were stained with Bodipy and visualized under fluorescence microscopy. White asterisks indicate partial autolysis of hyphae.

**Additional file 4.** *A. gossypii* strains used in this study. Table of *A. gossypii* strains used in this study.

**Additional file 5.** List of primers used in this study. List of primers used in this study.
