# Supplementary figures and images for: Utilization of xylose by engineered strains of Ashbya gossypii for the production of microbial oils
Source: Biotechnol Biofuels. 2017 Jan 3;10:3. doi: 10.1186/s13068-016-0685-9 (PMC5209892; doi:10.1186/s13068-016-0685-9)

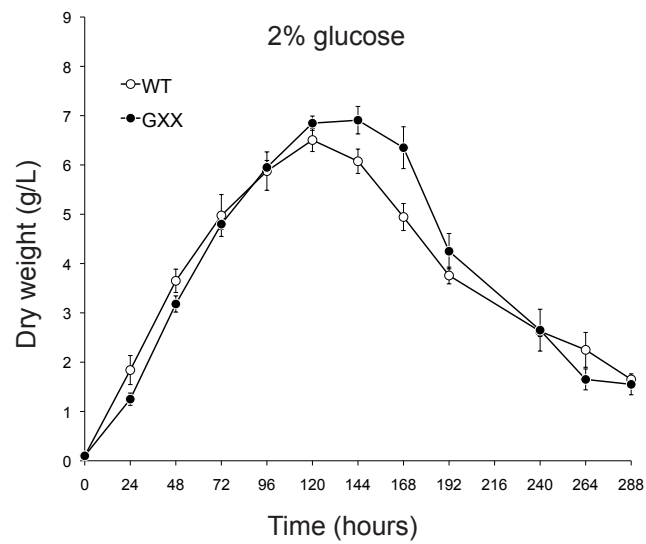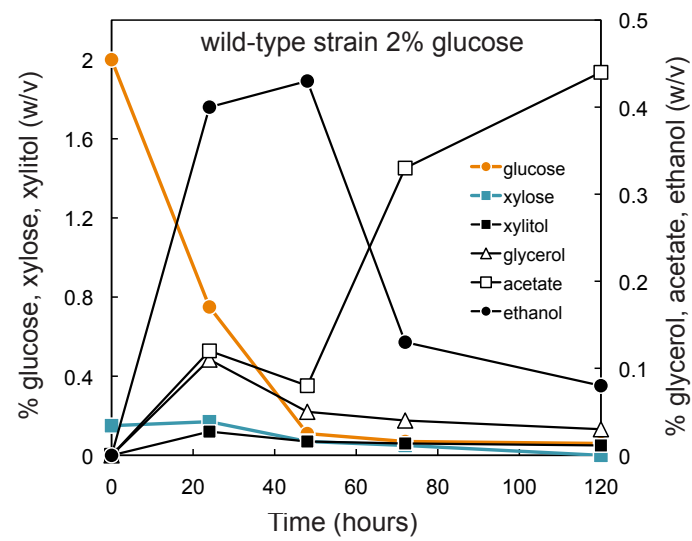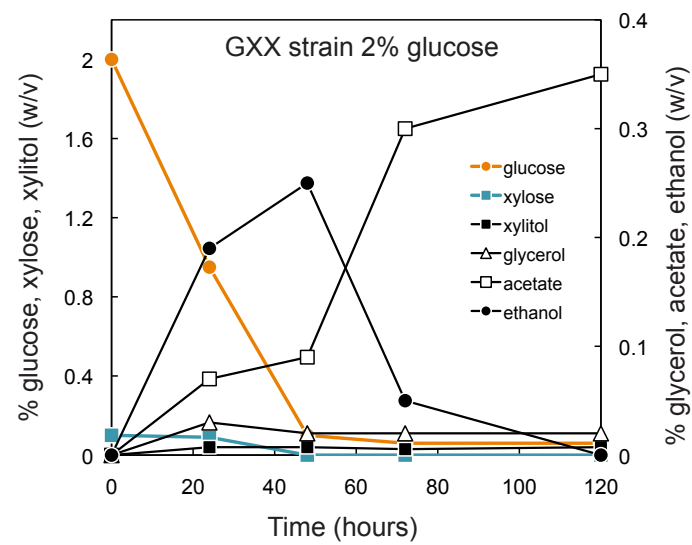

Supplement: Supplementary file 1 — Additional file 1. Utilization of glucose by the GXX strain of A. gossypii. Biomass production of the WT and GXX strains grown in MA2 medium with 2% xylose as the only carbon source (upper panel). Glucose consumption and metabolite production by the WT (middle panel) and GXX strains (lower panel) grown in MA2 medium with 2% glucose as the only carbon source. [file 13068_2016_685_MOESM1_ESM.pdf]

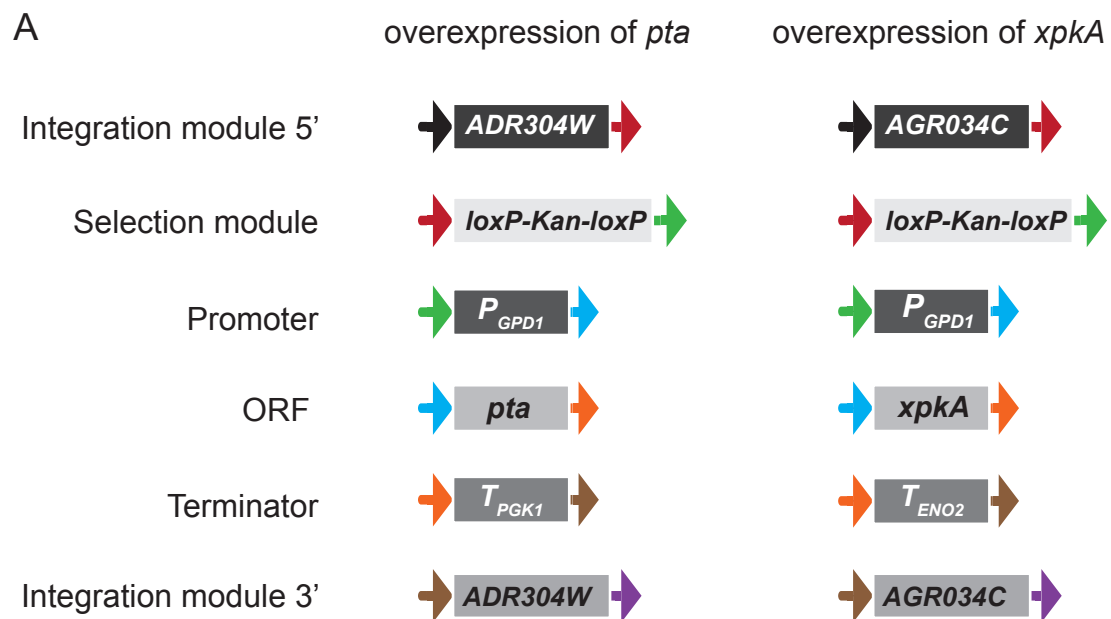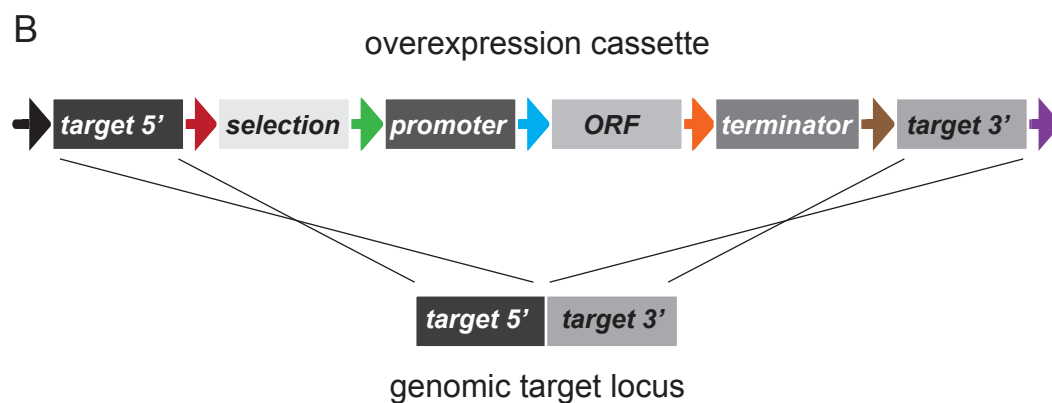

Supplement: Supplementary file 2 — Additional file 2. Cloning strategy for the overexpression of pta and xpkA genes. (A) Six modules were assembled for the construction of the overexpression cassettes: integration modules 5′ and 3′, selection module, promoter, terminator and the corresponding ORF (pta or xpkA). (B) The six modules were assembled following a one-pot DNA-shuffling (see “Methods” section) and the overexpression cassettes were integrated in the corresponding genomic loci (ADR304W for the pta cassette and AGR034C for the xpkA cassette). [file 13068_2016_685_MOESM2_ESM.pdf]

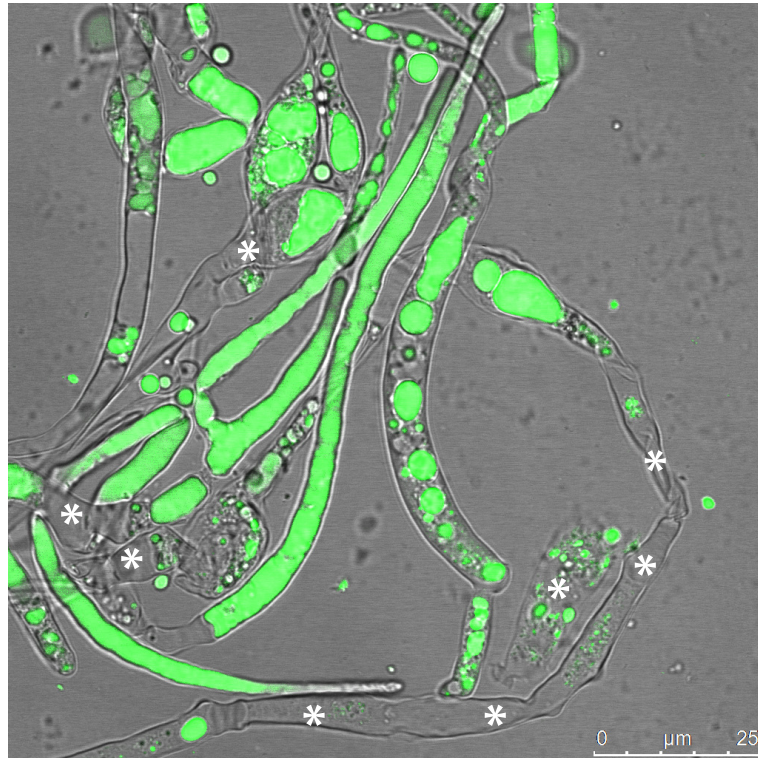

Díaz-Fernández et al., 2016. Additional File 3

Supplement: Supplementary file 3 — Additional file 3. Lipid bodies of the A. gossypii GXX-PX-ß∆ strain. Micrograph of the GXX-PX-ß∆ strain grown during 5 days in media containing 8% xylose plus 2% oleic acid. Lipid bodies were stained with Bodipy and visualized under fluorescence microscopy. White asterisks indicate partial autolysis of hyphae. [file 13068_2016_685_MOESM3_ESM.pdf]
