# Supplementary material for: Utilization of xylose by engineered strains of Ashbya gossypii for the production of microbial oils
Source: Biotechnol Biofuels. 2017 Jan 3;10:3. doi: 10.1186/s13068-016-0685-9 (PMC5209892; doi:10.1186/s13068-016-0685-9)
Supplement: Supplementary file 4 — Additional file 4. A. gossypii strains used in this study. Table of A. gossypii strains used in this study. [file 13068_2016_685_MOESM4_ESM.docx]

Additional File 4. *A. gossypii* strains used in this study.

| *Strain* | *Genotype* | | *Phenotype* | *Source* |
| --- | --- | --- | --- | --- |
| WT | | wild type (ATCC 10895) | WT | our lab stock |
| *A625* | | *loxP-KanMX4-loxP-P_GPD_-GRE3* | G418^R^ | this work |
| *A630* | | *P_GPD_-GRE3* | WT | “ |
| *A637* | | *P_GPD_-GRE3, loxP-KanMX4-loxP-P_GPD_-XKS1* | G418^R^ | “ |
| *A647* | | *P_GPD_-GRE3, P_GPD_-XKS1* | WT | “ |
| *A660* | | *P_GPD_-GRE3, P_GPD_-XKS1, loxP-KanMX4-loxP-P_GPD_-XYL2* | G418^R^ | “ |
| *A665 (****GXX****)* | | *P_GPD_-GRE3, P_GPD_-XKS1, P_GPD_-XYL2 (****GXX strain****)* | xyl^+^ | “ |
| *A673* | | *GXX, loxP-KanMX4-loxP-P_GPD_-pta* | G418^R^, xyl^+^ | “ |
| *A695* | | *GXX, P_GPD_-pta* | xyl^+^ | “ |
| *A725* | | *GXX, P_GPD_-pta, loxP-KanMX4-loxP-P_GPD_-xpkA* | G418^R^, xyl^+^ | “ |
| *A729 (****GXX-PX****)* | | *GXX, P_GPD_-pta, P_GPD_-xpkA (****GXX-PX strain****)* | xyl^+^ | “ |
| *A738(****GXX-PX-ß∆****)* | | *GXX, P_GPD_-pta, P_GPD_-xpkA, pox1Δ (****GXX-PX-ß∆ strain****)* | G418^R^, xyl^+^ | “ |
