# Supplementary material for: Utilization of xylose by engineered strains of Ashbya gossypii for the production of microbial oils
Source: Biotechnol Biofuels. 2017 Jan 3;10:3. doi: 10.1186/s13068-016-0685-9 (PMC5209892; doi:10.1186/s13068-016-0685-9)
Supplement: Supplementary file 5 — Additional file 5. List of primers used in this study. List of primers used in this study. [file 13068_2016_685_MOESM5_ESM.docx]

Additional File 5. List of primers used in this study.

| *Primer* | *Sequence* | *Purpose* |
| --- | --- | --- |
| loxPMK-*P_GPD_*-*GRE3*-fw | 5’-GGC GCT TTC GAA TAC CCT ATT TGC TTT GCA TTC CCC TTC TAG AGC ATG TTC ATG CAC GGC TGA CCG TGT CAA CCT GCT GAC GGA TCC CCG GGT TAA TTA A-3’ | *GRE3* overexpression |
| loxPMK-*P_GPD_*-*GRE3*-rv | 5’-ACC ATT GCG ATG TCC TGG CCT GTA ATG CAC CTT GAT AAA TCT GGT GGT GTT CCA CGC ATA TAC AAC TGC TTA TAT ACC ATT GTG CGG TGT GTA TGT GTG G-3’ | *GRE3* overexpression |
| loxPMK-*P_GPD_*-*XYL2*-fw | 5’-GGT TGC ACT TGG CAT ATG CGG AAG CGT CAT CTA CAA ACA CCG GCG TAT TTA GCA CAC TGT CAC ACT AGC ATC AAT AAG TAC GGA TCC CCG GGT TAA TTA A-3’ | *XYL2* overexpression |
| loxPMK-*P_GPD_*-*XYL2*-rv | 5’-ATA ACT GGG ACG GGC CTG TGT TCA TAC ACT ATA GCC CCC TTC TCG CGG AGG ACA ACC GCC TGC TGC ATA CTT GAT GAC ATT GTG CGG TGT GTA TGT GTG G-3’ | *XYL2* overexpression |
| loxPMK-*P_GPD_*-*XKS1*-fw | 5’-TAG GCA GTG CCG CCG CGA CCA CCA AAG ATT TAT AGT GCA GTA TGT AAT TCA CTC GGT ATA AAA TTA AGG AGA TGC CAG GGC GGA TCC CCG GGT TAA TTA A-3’ | *XKS1* overexpression |
| loxPMK-*P_GPD_*-*XKS1*-rv | 5’-GCC AGG TCC TCG TCG ATG GCA AGG CAC TTC AAC TGT TGC GTT GAC AGA TCG AAC CCC AAG TAC AGC TTG CTT TCC GCC ATT GTG CGG TGT GTA TGT GTG G-3’ | *XKS1* overexpression |
| *(GATG)-pta-fw* | 5’-TTT GGT CTC GGA TGG CAG ATT TAT TTT CAA CAG TGC A-3’ | *pta* overexpression |
| *(TAGT)-pta-rv* | 5’-TTT GGT CTC TAC AGT GCT TGC GCC GCT-3’ | *pta* overexpression |
| (GATG)-*xpkA-fw* | 5’-TTT GGT CTC AGA TGC CGC GGG AAG TGA TT-3’ | *xpkA* overexpression |
| (CTAA)-*xpkA-rv* | 5’-TTT GGT CTC ATT AGT TAA ACG ACG GCA TGT TAT AGG-3’ | *xpkA* overexpression |
| *POX1∆*-fw | 5’-GGC AGC TAG CGG TGG CTA TAA AGA GCG GGA AAA AGC AGA GCG TAG CAG CAG CCA AGG ATC GAC AAG ATG CGG ATC CCC GGG TTA ATT AA-3’ | *POX1* deletion |
| *POX1∆*-rv | 5’-GTA ATA TCA GCG AGC AGA GAT AGA CTC AAC TAC TTG CCA AGG TTC TTG AAG GAG GTC TCG CTG ATG TTC AGA ATT CGA GCT CGT TTA AAC-3’ | *POX1* deletion |
| *GRE3*-a | 5’-ACA TAC TGC GTG TCC GTG TT-3’ | *Analytical PCR* |
| *XYL2*-a | 5’-ATT GCC AGT ACG CTC AGG AC-3’ | *Analytical PCR* |
| *XKS1*-a | 5’-GAA CCC AAT GTA CCC TGC CA-3’ | *Analytical PCR* |
| POX1-a3 | 5’-GCC CGG GTA ACC AAA CCT TA-3’ | *Analytical PCR* |
| KanB1 | 5’-CTG CAG CGA GGA GCC GTA AT-3’ | *Analytical PCR* |
| *GRE3*-RT-fw | 5’-GGC TCG GGT GCT GGA AGA TT-3’ | *GRE3* qRT-PCR |
| *GRE3*-RT-rv | 5’-CAG CGG GAA GTG GAT GTA GAA C-3’ | *GRE3* qRT-PCR |
| *XYL2*-RT-fw | 5’-TCG GGC TGG GCT GCT TTT AGT-3’ | *XYL2* qRT-PCR |
| *XYL2*-RT-rv | 5’-AGC GTG CCC TCC AGT GTC AGA TA-3’ | *XYL2* qRT-PCR |
| *XKS1*-RT-fw | 5’-ACA CAG GCC CGT CCC AGT TA-3’ | *XKS1* qRT-PCR |
| *XKS1*-RT-rv | 5’-GCA TCT CGC GAC ACA GGT TG-3’ | *XKS1* qRT-PCR |
| *UBC6-fw* | 5’-CAA CGA TAC TGA CTG GGC TGC TAA-3’ | *UBC6*  qRT-PCR |
| *UBC6-rv* | 5’-GGC GCG TAT CCT ATC CTC TGG-3’ | *UBC6*  qRT-PCR |
| *pta_fwd_qPCR* | 5’-CCC GGC TCT TCA AAT CAT CAA AA-3’ | *pta*  qRT-PCR |
| *pta_rev_qPCR* | 5’-AAT TCG CCG TCA AGT GTC AGT TCA-3’ | *pta*  qRT-PCR |
| *xpkA_fwd_qPCR* | 5’-GCA CGC CGA AAG GTT GGA GT-3’ | *xpkA*  qRT-PCR |
| *xpkA_rev_qPCR* | 5’-CAC GGT ATG CGG GTT TTT CAC A-3’ | *xpkA*  qRT-PCR |
